# Inhibition of cross-reactive carbohydrate determinants (CCDs) enhances the accuracy of in vitro allergy diagnosis 

**DOI:** 10.5414/ALX01638E

**Published:** 2017-08-04

**Authors:** W. Aberer, F. Holzweber, W. Hemmer, L. Koch, D. Bokanovic, W. Fellner, F. Altmann

**Affiliations:** 1 University Clinic of Dermatology, Medical University of Graz, Austria; 2 Medical Laboratory, Villach, Austria; 3 loridsdorf Allergy Center, Vienna, Austria; 4 Kwizda Pharma, Vienna, Austria; 5 Department of Chemistry, University of Agricultural Sciences and Natural Resources, Vienna, Austria

**Keywords:** cross-reactive carbohydrate determinants, CCD, allergy diagnosis, CCD-blocker, CCD-inhibition

## Abstract

Background: Cross-reactive carbohydrate determinants (CCDs) as they occur on natural allergens from plants and insects influence the measurement of antigen-specific IgE-antibodies in the context of *in vitro* allergy diagnosis. When positive results are based solely on the reaction of CCDs with anti-CCD IgE, results must be rated as false-positive. A generally applicable solution to this problem has not yet been presented. Methods/Patients: Sera of patients for whom an assumed allergy should be verified or ruled out were tested with three methods for specific IgE determination (a multiallergen teststrip format, a single allergen test and an allergen-component array) in the absence and presence of a novel, semi-synthetic CCD-blocker. The study was not prospective and for many patients unequivocal clinical data were missing; the data section thus focusses on few, well-defined patient sera. Results: More than 20% of all patients were tested positive for IgE-anti-CCD antibodies and hence against a multitude of similarly glycosylated allergen extracts in a strip-based multiallergen test. Incubation of these positive sera with the CCD-blocker led to significant reductions of read-out values and in many cases to negative test results. The inhibitory efficiency was highest for the allergen strip test and for the component array. Results remained positive for relevant allergens for which a true sensitization had been indicated by skin tests or other means. The CCD-blocker did not alter the read-outs for unglycosylated allergens or – with CCD-negative sera – for all allergens. Conclusion: Elimination of CCD-specific IgE antibodies by means of a synthetic CCD-blocker drastically reduced the number of false-positive *in vitro* test results without compromising the sensitivity for relevant IgE interactions. Thus, the herein described CCD-blocker constitutes a valuable tool for increasing the test specificity of routine *in vitro* allergy diagnosis.

German version published in Allergologie, Vol. 37, No. 2/2014, pp. 46-54

## Introduction 

Specific IgE antibodies to cross-reactive carbohydrate determinants (CCDs) in the serum of persons with pollen allergy are responsible for cross-reactivity with several inhalant and food allergens as well as insect venom and latex [1, 2, 3, 4, 5, 6, 7, 8]. Conversely, insect venoms are potent inducers of CCD-specific IgE antibodies. Therefore, positive *in vitro *reports for pollen, food and latex are commonly found in non-atopic persons with insect venom allergy [9, 10]. These CCD antibodies are of little or no clinical relevance [2, 4, 5, 6, 11], but may be a potential reason for false-positive test results and inappropriate clinical consequences. Based on such test results, atopic children may be given unsuitable dietary instructions or meaningless prohibitions and corrective measures, or the clinician institutes unjustified immunotherapy [12]. The presence of anti-CCD IgE can be demonstrated with the aid of a screening allergen containing CCD, such as MUXF^3^, or by means of natural glycoproteins such as bromelain, peroxidase from horseradish, or ascorbate oxidase. A positive test merely confirms the presence of such antibodies in serum, but does not permit a reliable statement as to whether the reactivity with a specific allergen source is exclusively due to CCDs or whether protein-specific IgE antibodies are additionally involved. The AWMF guidelines known as *In vitro Allergiediagnostik (In vitro allergy diagnosis)* [13] state that an inhibition test with the specific CCD screening allergen can markedly enhance the specificity of the test. In the references mentioned therein [14, 15], the inhibition test is regarded as a desirable tool to enhance specificity, but specific recommendations are not provided. Data in this regard are lacking and the technical application remains unresolved. 

Thus, there is a need for a simple procedure to separate the chaff of anti-CCD IgE antibodies from the wheat of clinically relevant antigen-specific IgE. 

Irrelevant or false-positive test results should be suppressed while relevant ones should not be influenced. The technical handling of the procedure should be simple, the substance stable and unproblematic, the costs low, the results reproducible, and the validity high. 

A CCD blocker to resolve exactly this problem was developed recently (www.proglycan.com). In the present article we describe the application of this CCD inhibitor using three different measuring systems for allergen-specific IgE. 

## Methods 

### Patients and serum samples 

At a referral laboratory, all serum samples received for sIgE testing from July 2011 to December 2012 were tested for the presence of anti-CCD IgE using the multi-allergen strip test named AllergyScreen, provided by Mediwiss Analytic Company (Moers, Germany). The samples had been sent for investigation of suspected inhalant and/or food allergies and insect venom allergy. From January 2012 onward, all samples showing evidence of IgE antibodies to CCDs were re-tested with inhibition by pre-treatment with the CCD (final concentration 20 μg/ml) in order to compare the presence of specific IgE antibodies to inhalant or food allergens with and without the blocker. Both results were sent to the referring physician or site with appropriate comments. 

The validity of the study is limited by the fact that, for the majority of samples, the patients’ symptoms were not known with certainty at the laboratory. Therefore, only random samples could be used to make statements concerning the frequency of anti-CCD antibodies and their susceptibility to blocking. 

## CCD blocker 

**Figure 1.**
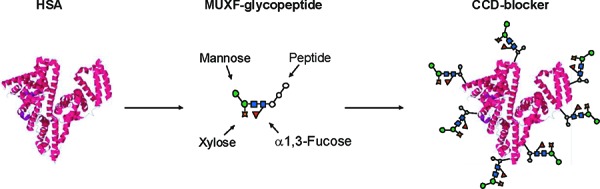
Production of the CCD blocker from human serum albumin (HSA) and the MUXF glycopeptide.

The CCD blocker is a synthetic glycoprotein made from human serum albumin (HSA, Sigma-Aldrich) and a highly purified vegetable glycopeptide. Bromelain is first isolated from pineapple stem extract (Sigma-Aldrich) and digested with a protease. Any existing protein epitopes are destroyed by this process. The glycopeptide thus obtained is purified with the so-called MUXF structure (specifically MUXF³) (Figure 1) and a dipeptide or tripeptide in order to achieve homogeneity. Identity and purity are tested by MALDI-TOF mass spectroscopy. The MUXF glycopeptide is coupled to HSA via dinitrodifluorobenzene (Sigma-Aldrich). MALDI-TOF mass spectroscopic analysis revealed the moderate presence of at least nine MUXF glycopeptides. We found one neo-glycoprotein which could not contain any epitopes of vegetable proteins because of the manufacturing procedure, and the only protein component was contained human HSA; this distinguishes it from its precursor variations [3]. As the CCD blocker itself contains no antigen determinants, one may, in all probability, rule out the risk of unintentional suppression of relevant IgE allergen reactions. The polyvalence of the CCD blocker ensures its high efficacy and, consequently, its low working concentration. 

The CCD blocker can be added to sera before determination of specific IgE antibodies using any type of IgE testing system. Based on dilution series and experiments in the ELISA format with a pooled serum sample containing ascorbate oxidase, a final concentration of 20 mg/l was selected. This corresponds to the addition of 10 µl of the CCD inhibitor (= 10 µg) to 0.5 ml of serum. Subsequent experiments showed that markedly lower concentrations achieve sufficient inhibition in a large majority of serum samples. Pre-incubation before further use of the serum is not required. The ready-to-use CCD blocker solution was divided into portions, deep-frozen at –20° C, defrosted, and could be used for 4 weeks thereafter at 4 – 8°C. The final preparation is equivalent to the ProGlycAn CCD blocker described in the Internet (www.proglycan.com). 

## Determination of sIgE 

Serum samples were tested for the presence of IgE antibodies using three commercially available test procedures. The Mediwiss multi-allergen strip test was used for the large majority of samples. Two strips were used for adults, i.e. one for inhalant (18 allergen extracts) and one for food allergens (14 plant and animal foods). In children we used a mixed panel (inhalant and food allergens). The specially prepared strips contained pathways for CCD allergens (horseradish peroxidase, ascorbate oxidase, bromelain or a mixture of the three). Alternatively, or additionally, single-allergen testing was performed with ImmunoCAP of ThermoFisherScientific (previously Phadia). Anti-CCD IgE was determined here using the CCD test allergen MUXF3 (o214). The serum samples were divided and then subjected to the respective IgE testing method either in untreated form or together with the CCD inhibitor (the final concentration was 20 mg/l) without preincubation. Some serum samples were subjected to component-specific diagnostic investigation using ImmunoCAP ISAC (ThermoFisherScientific). 

## Results 

### Preliminary experience 

Of approximately 6,000 serum samples tested in 2012, which were investigated with the multi-allergen strip test (inhalant and/or food allergens), 22% showed IgE antibodies to CCD’s; 86 % of these had anti-CCD IgE antibodies of RAST classes 2 or higher. Classification of positive CCD reports according to age showed maximum levels in the age group of 10- to 20-year-old persons (35% were affected). On the strip test for inhalant as well as food allergens, these serum samples revealed a number of clearly positive signals, which were by no means demonstrably related to the patients’ medical history. In order to achieve a meaningful laboratory diagnosis for these patients as well, we started to use the synthetic CCD blocker. In many CCD-positive serum samples, pre-incubation with the CCD blocker resulted in the complete disappearance of many bands or at least a drop in signals to below the reference value. 

In a random sample of 43 fully evaluable patients (CCD’s present, all clinical data concerning the patients were verified), complete inhibition of CCDs as well as specific allergens was achieved in 18 (41.9%). In 22 patients (51.2%) the inhibition was partial (thus no unequivocal answer, but one could view the situation more specifically). In 3 patients (7%) the inhibition failed (CCD reduced; IgE antibodies to specific allergens unchanged). As the study was not prospective, and a large part of the clinical data could not be verified, a general evaluation of data according to these criteria was not performed. This will be tested in the exploratory phase of the system and presented on the basis of unequivocal patient data. Encouraged by the prospect of being able to eliminate a large number of irrelevant reactions by means of the CCD blocker, a few cases were subjected to more exact testing with various IgE test methods while taking clinical reports into account. 

### Case reports 

**Figure 2.**
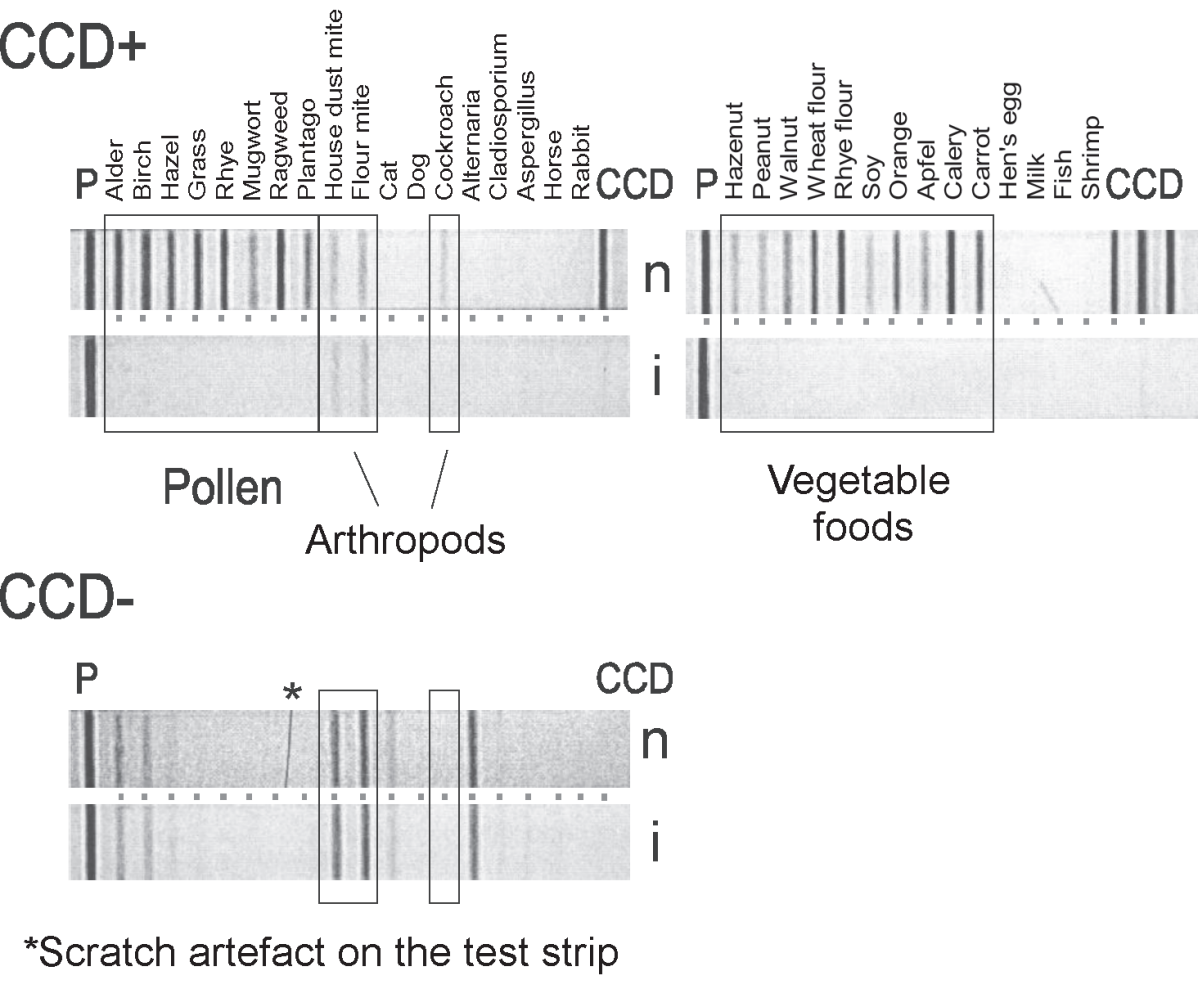
Effect of the synthetic CCD blocker. Results for patients with anti-CCD IgE antibodies and one without such cross-reactive IgEs, as obtained on the Mediwiss test strip without (**n**) and with (**i**) an inhibitor. The left strips contained inhalant allergens (P, positive control; CCD, mixture of bromelain, horseradish peroxidase and ascorbate oxidase). Vegetable and animal foods as well as bromelain, horseradish peroxidase and ascorbate oxidase were applied on the right strip as CCD indicators. The symptoms of the CCD-positive patient were confined to perennial nasal congestion.

The case of a 16-year-old girl serves as a typical example (Figure 2). Without inhibition her serum tested positive for pollen, dust mite, cockroach, and all vegetable foods. When the test was performed with CCD inhibition, all bands with the exception of those for dust mites disappeared. This outcome concurred with the patient’s clinical symptoms. 

The case of a 70-year-old woman was even more convincing. Her serum tested strongly positive for a large number of vegetable allergens on the test strip, but her skin-prick test was not positive for any pollen extract (Table 1, w 70). On CCD inhibition all tests were negative. Several hundreds of similar findings and cases could be shown here. A few are listed in Table 1. The inhibited outcomes provide a clear view of presumably relevant allergens. In the following we will present a case subjected to specific further analyses. 

The serum of a 46-year-old man was sent for investigation of sensitization, although he had no identifiable symptoms of allergy (the clinical diagnosis was sinobronchial syndrome). A bee or wasp venom allergy was also suspected. The serum was first tested on a strip test, which showed wide polysensitization to plant allergen extracts. Of 25 strongly positive reactions (especially those for pollen), none remained positive after inhibition (Table 2). 

These unequivocal results clearly raise the question as to whether, and to what extent, the CCD blocker suppresses reactions that may actually indicate relevant sensitization. We also wished to determine whether this type of CCD inhibition could be used in other test procedures as well, especially ImmunoCAP and ISAC. A notable aspect of the non-inhibited values are the much more moderate data in regard of sIgE concentrations, which were retained on ImmunoCAP when compared to the semiquantitatively treated multi-allergen strip test. Nevertheless, inhibition below the threshold value was only achieved in 5 of 8 cases. Especially the grass mixture and bee venom remained very high (above 1 kU/l). In the case of bee venom, however, it is not certain whether this was a protein-based cross-reaction because the patient was markedly sensitized to wasp venom. Reactions to wasp venom as well as its components remained clearly positive even with the CCD inhibitor. The MUXF3 test allergen was not measured in this patient, but was measured in a few others. We observed partial inhibition in many cases. Accordingly, the mild persisting reaction to grass or ragweed pollen may still be rated false-positive, especially because all grass components and Art v 1 tested negative on ISAC. One exception was nPhl p4, which is a natural component and apparently tested positive here in the non-inhibited approach because of its CCD glycans. This is also true of a few other natural components. Since an insect venom allergy was suspected in this patient, CAP and ISAC tests for the venom and its components were performed. A rather confusing aspect is the discrepancy of reactions to rApi m1 on the ImmunoCAP and the ISAC, and the apparent inhibition of this recombinant allergen on ImmunoCAP by the CCD blocker. As IgE binding to rApi m1 was not influenced by the blocker in some cases (data not shown), we regard this doubly questionable test outcome (*i.e*. the positive CAP value to rApi m1) as an unexplained artifact. It is more important to note that a few recombinant allergen components were measured on ImmunoCAP and ISAC in the presence of a CCD inhibitor as well. The results show that the inhibitor caused no undesirable changes apart from statistical fluctuations. 

## Discussion 

Although the CCD problem has been known for a long time [4, 6, 16], it has been largely ignored in clinical practice. However, this problem must be confronted when using multi-allergen strip tests (as we did in the present study), failing which it would be impossible to perform a meaningful evaluation in about one fourth of allergy patients. This is in contrast to a number of frequently used methods for sIgE determination, by which only specific allergens are tested selectively on the basis of preliminary information. Thus, a positive report does not arouse any suspicion, regardless of whether it is clinically correct or false. The hope in regard of recombinant allergen components is justified because these are produced without CCD [17]. It should be noted that component tests such as ImmunoCAP ISAC are basically also influenced by the CCD problem because they contain a few natural glycosylated components (see Table 2). Besides, in the course of routine diagnostic investigation, while consistently giving preference to individual components, one is still dependent on the total extracts because all relevant sources of allergens (especially those of foodstuffs) are by no means adequately accounted for by the components. 

When using the more economical tests with allergen extracts, one must eliminate clinically irrelevant CCD reactions when testing for inhalant, food, latex and insect venom sensitization. In the present investigation with IgE tests of various designs, we show that CCD-based reactions are usually below the reference value, but are always clearly reduced in all cases. On the other hand, protein-based reactions, considered relevant, remain unimpaired. As the required volume of the reagent is only 2% of the investigated serum, even in cases of marginally positive baseline IgE values (class 1) to a specific allergen the loss of sensitivity due to dilution of the sample is negligible. Binding to recombinant CCD-free allergens persists in the presence of a CCD blocker, thus corroborating its specificity (refer, for instance, to the ISAC values of Patient m 12 in Table 2). 

In specific cases the CCD blocker tested here failed to achieve sufficient inhibition. This was especially true of a few ImmunoCAP tests, such as those for grass, common ragweed, or insect venom, and appears to correlate with the level of anti-CCD IgE. Although the relevant allergen is clearly identified even in cases of incomplete inhibition in the presence of apparent double sensitization to insect venom (see Patient m 46 in Table 2), further investigations must be performed to determine whether raising the inhibitor concentration would be meaningful or necessary, and whether the slightly higher values despite inhibition were caused by factors other than CCDs. It should be mentioned that the results of bee venom testing did not entirely meet our expectations, but a comparison of inhibited and non-inhibited values with those for wasp venom does permit a clear decision as to what venom one should use for immunotherapy. 

The fact that not all relevant questionable reports could be investigated by CCD inhibition was because CCDs constitute just one potential source of error in *in vitro* allergy diagnosis. Several other factors lurk in their vicinity, such as poor IgE specificity, competition from IgE or other “mimickers of allergy” [2]. However, the present study does clearly show that the CCD problem is, quantitatively speaking, the prime cause of discrepant allergy reports. 

In the ideal case the clinician should be able to justifiably assume that all IgE antibodies remaining after inhibition are actually directed towards peptide epitopes and hence potentially of clinical relevance. This is undoubtedly a high demand of a test and may not always be achievable in actual practice. This demand of a CCD blocker appears to be exaggerated in view of the substantial divergence of various sIgE test procedures, regardless of the CCD problem. However, it may be stated that a breakthrough has been achieved here with relatively simple resources and without significant technical effort. The entirely justified recommendation to use a flow chart for obtaining diagnostic evidence of anti-CCD IgE in serum, described as a desirable measure in a recent overview, is quite difficult to put into practice in clinical routine – which includes the medical office and the laboratory for routine diagnostic investigation [18]. 

Prospective studies in specifically defined patient populations using different IgE test systems will have to follow now. Basically CCD inhibition should be investigated in test methods based on *in vitro* antibody binding. However, we have data to show that, in persons with an insect venom allergy, the basophil activation test may be disturbed by anti-CCD IgE [2]. 

We regard the present data – of which only a part have been shown here – as sufficient to establish that, even now, the mere suspicion of false-positive in vitro IgE reports should be followed by a subsequent test using an CCD inhibitor. The Mediwiss strip test and the ISAC multi-component array may even be generally used for the initial analysis. An official recommendation as to whether the CCD blockade should be routinely used in the future with some or even all methods in order to avoid such results from the very start will obviously require a broad-based prospective investigation. The results obtained with this CCD blocker might explain why different IgE test methods systematically yield diverse results and the products of the various manufacturers are therefore not comparable – a point of criticism that has been expressed for several decades now, but has received little attention from manufacturers thus far [19]. 

**Table 1.** Diagnostic data obtained an the multi-allergen test strip without (“MW std.” column) and with (“MW inhib.” column) CCD inhibition. In a few patients we performed additional skin tests (“SPT” columns).

Patient:	w 70			w 24			m 12		w 35		w 7		w 63	
Test:	MW std	MW inhib.	SPT	MW std.	MW inhib.	SPT	MW std.	MW inhbi.	MW std.	MW inhbi.	MW std.	MW inhbi.	MW std.	MW inhbi.
Inhalative														
Alder pollen	16.5	0	neg	3.6	0	neg	10.7	0	8.7	0	1	0	14.4	15.7
Birch pollen	30.5	0	neg	5.8	0	neg	19	0	7	0	1	0	14.4	15.9
Hazel pollen	26.9	0	ne	6.5	0	neg	25.7	0	10.4	0	1.6	0	12.2	7.1
Grass mix	23.8	0	neg	28	19.7	pos	21.2	0	4.8	0	0.6	0	4.4	0
Rye pollen	53.4	0.2	neg	>100	9.4	very pos	>100	0	8.5	0	1	0	7.7	0
Wormwood pollen	7.2	0	neg	2.2	0	neg	6.5	0	1.9	0	0	0	1.1	0
Ragweed pollen	>100	0.2	neg	23	0	neg	81	0	16.7	0.4	3.3	0	12	0.3
Ribwort plantain pollen	13.9	0	neg	5.1	0	neg	9.3	0	4.7	0	0.7	0	1.7	0
Cockroach	10.2	0		2	0	neg	18.4	19.6	3.4	0	0.3	0	0.6	0
Houst dust mite	0	0	neg	0	0	neg	4.9	7.3	0.8	0.7	4.4	3.3	0	0.3
Flour mite	0	0	neg	0	0	neg	6.1	7.6	1.4	0.9	3.7	2.4	0	0
Cat	0	0	neg	0	0		4	5.9	18.4	21.5	0	0	1.3	9.9
Dog	0	0		0	0		0.6	0.6	0	0	0.2	0	3.5	5
Alternaria t.	0	0	neg	0	0	neg	0	0	0	0	0	0	0	0
Cladosp. h.	0	0	neg	0	0	neg	0	0	0	0	0	0	0	0
Aspergillus f.	0	0	neg	0	0	neg	0	0	0	0	0	0	0	0
Horse	0	0		0	0	ne	0	0	0	0	0	0	0.2	0
Rabbit	0	0		0	0	neg	0	0	0	0	0	0	0	0
CCD mix	26.1	0	n. a.	4.5	0	neg	22.8	0	4.3	0.4	3.5	0.3	2.7	0
Food allergen strip:														
Hazelnut	3.4	0					6.3	0	2.3	0				
Peanut	1.7	0					5.5	0	0.8	0				
Walnut	6.4	0					9.6	0	3.4	0				
Wheat flour	42.3	0					25.1	0	7.9	0				
Rye flour	73.1	0					53	0	16.8	0				
Soy	1.4	0					5	0	0.5	0				
Orange	4	0					10.9	0	1.7	0				
Apple	1.8	0					5.3	0	1.3	0				
Celery	27.1	0					>100	0	6.4	0				
Carrot	14.3	0					20.5	0	5.3	0				
Chicken egg	0	0					0	0	0	0				
Milk	0	0					0	0.3	0	0				
Cod	0	0					0	0	0	0				
Shrimp	0	0					5.7	4.3	0	0				
Bromelain	38.4	0					98	0	0.6	0				
Horseradish peroxidase	9.3	0					64	0.15	5.2	0.4				
Ascorbin oxidase	30.4	0					92.6	0	10.3	0				

**Table 2:** Results for patient “m 46“, investigated with the multi-allergen strip (MW), ImmunoCAP and ImmunoCAP ISAC without (n) and with (i) CCD inhibition. “A” indicates anticipated inhibition or non-inhibition that was rated correct (“C”). Questionable results are marked by the (×) sign.

	MW n	MW i		CAP n	CAP i			ISAC n	ISAC i	
Allergen source	U/mL	U/mL		U/mL	U/mL		Component	ISU-E	ISU-E	
Birch pollen	> 100	0	A	19.6	0.11	↑	rBet v 1 / v2 / v4	0	0	
Grass pollen	99.7	0	A	25.0	1.11	×	rPhl p1/ 2/ 5/ 6/ 7/ 11/ 12	0	0	
Rye pollen	> 100	0	A				nPhl p4*/nCyn d 1*	10/26	0/0	↑
Wormwood pollen	14.9	0	A	21.6	0.21	↑	nArt v1	0	0	
Ragweed pollen	> 100	0	A	25.7	0.49	×	nAmb a1	0	0	
Ribwort plantain pollen	32.9	0	A				rPla l 1	0	0	
House dust mite	0.59	0	A				rDer p 1/nDer p2	0	0	
Cockroach	13.6	0	A				rBla g 1/2/5/7	0	0	
Cat	0	0					rFel d 1	0	0	
Dog	0.37	0					rCan f 1	0	0	
Hazelnut	3.4	0	↑	17.4	0.08	↑	rCor a 1/8	0	0	
Peanut	1.7	0	↑				rAra h 1/2/3/8/9	0	0	
Walnut	6.4	0	↑				nJug r 1/2*/3	0/10/0	0/0/0	↑
Wheat flout	42.3	0	↑				rTri a 14/19	0	0	
Soy	1.4	0	↑				rGly m 4/nGly m 5/6	0	0	
Apple	1.8	0	↑				rPru p 1/3	0	0	
Celery	27.1	0	↑				rApi g 1	0	0	
Bee venom				28.5	1.1	×				
rApi m1				1.63	0.31	↑	rApi m1	0	0	↑
Wasp venom				28.5	18.3	C	rPol d5	1.0	1.5	→
rVes v1 / v5				11.8/48.7	9.7/44.6	→	rVesv5	6.2	8.9	→
CCD mix	> 100	0	↑				CCD MUXF3	20	0	↑
CCD bromelain	38.4	0	↑				nCry j 1*	9.3	0	↑
CCD horseradish peroxidase	9.3	0	↑				nCup a 1*	11	0	↑
CCD ascorbate oxidase	30.4	0	↑				nOle e 1*	4.5	0	↑
							nPla a 1*	12	0	↑
